# Factors influencing malaria control policy-making in Kenya, Uganda and Tanzania

**DOI:** 10.1186/1475-2875-13-305

**Published:** 2014-08-08

**Authors:** Clifford M Mutero, Randall A Kramer, Christopher Paul, Adriane Lesser, Marie Lynn Miranda, Leonard EG Mboera, Rebecca Kiptui, Narcis Kabatereine, Birkinesh Ameneshewa

**Affiliations:** Centre for Sustainable Malaria Control and School of Health Systems and Public Health, University of Pretoria, Private Bag 323, Pretoria, 0001 South Africa; International Centre of Insect Physiology and Ecology, P.O. Box 30772, Nairobi, Kenya; Nicholas School of the Environment, Duke University, 9 Circuit Drive, Durham, NC 27708 USA; Duke Global Health Institute, Duke University, 310 Trent Drive, Durham, NC 27710 USA; School of Natural Resources and Environment, University of Michigan, 440 Church Street, Ann Arbor, MI 48109 USA; National Institute for Medical Research, 2448 Barack Obama Drive, Dar es Salaam, P.O. Box 9653, Tanzania; Division of Malaria Control, Ministry of Health, P.O. Box 20750, Nairobi, Kenya; Vector Control Division, Ministry of Health, Kampala, P.O. Box 1661, Uganda; WHO Regional Office for Africa, P.O. Box 06, Brazzaville, Congo Republic

**Keywords:** Malaria, Policy makers, Decision-analysis tools, MDAST, Multi-sectoral approach

## Abstract

**Background:**

Policy decisions for malaria control are often difficult to make as decision-makers have to carefully consider an array of options and respond to the needs of a large number of stakeholders. This study assessed the factors and specific objectives that influence malaria control policy decisions, as a crucial first step towards developing an inclusive malaria decision analysis support tool (MDAST).

**Methods:**

Country-specific stakeholder engagement activities using structured questionnaires were carried out in Kenya, Uganda and Tanzania. The survey respondents were drawn from a non-random purposeful sample of stakeholders, targeting individuals in ministries and non-governmental organizations whose policy decisions and actions are likely to have an impact on the status of malaria. Summary statistics across the three countries are presented in aggregate.

**Results:**

Important findings aggregated across countries included a belief that donor preferences and agendas were exerting too much influence on malaria policies in the countries. Respondents on average also thought that some relevant objectives such as engaging members of parliament by the agency responsible for malaria control in a particular country were not being given enough consideration in malaria decision-making. Factors found to influence decisions regarding specific malaria control strategies included donor agendas, costs, effectiveness of interventions, health and environmental impacts, compliance and/acceptance, financial sustainability, and vector resistance to insecticides.

**Conclusion:**

Malaria control decision-makers in Kenya, Uganda and Tanzania take into account health and environmental impacts as well as cost implications of different intervention strategies. Further engagement of government legislators and other policy makers is needed in order to increase funding from domestic sources, reduce donor dependence, sustain interventions and consolidate current gains in malaria.

## Background

Malaria ranks high among the major infectious diseases undermining health and socio-economic development in Africa [1, 2]. While a scaling up of interventions including vector control and treatment has led to a significant decline in the disease on the continent during the last decade, the gains are fragile and the control efforts need strengthening [3]. Uncertainties abound regarding the present achievements in malaria control due to various factors such as current widespread vector resistance to a range of insecticides normally incorporated in protective mosquito nets and also used for indoor residual spraying (IRS) [4]. Perhaps even more worrying for Africa is the looming threat of malaria parasite resistance to current first-line drugs containing the compound artemisinin. Artemisinin-based combination therapy (ACT) has been the most efficacious drug against malaria parasites that are resistant to the previously commonly used and relatively cheaper drugs, most notably, chloroquine and sulphadoxine-pyrimethamine [5]. Resistance to artemisinin has been recently detected in four countries of South-East Asia including Cambodia, Myanmar, Thailand and Vietnam [6]. Any further westward spread of artemisinin resistance to the more malaria-endemic regions in India and sub-Saharan Africa could have serious consequences due to a possible resurgence of the disease in countries where it has been on the decline [7]. From a funding point of view, doubts exist regarding sustainability of the current levels of international support for malaria control due to economic difficulties facing some of the traditional western donor countries [8]. Similarly, malaria is now known to be intricately linked to poor socio-economic conditions prevalent among many developing countries especially in Africa [9].

In view of malaria’s complex epidemiological and socio-economic dimensions, policy decisions regarding its control are often difficult to make at a national level as they have to carefully consider and respond to the needs and circumstances of a large number of stakeholders [10] within a resource-constrained setting. Choosing different vector and disease control options may require making difficult tradeoffs among competing health, economic and, in certain cases, environmental objectives. A case in point is the dilemma facing several African countries and the international malaria community regarding whether or not to use the insecticide dichloro-diphenyl-trichloroethane (DDT) for IRS [11, 12]. While spraying with DDT continues to be used as a primary vector control intervention by certain countries [13–15], concerns about its potential negative health and environmental impacts have led to continued calls for its ban worldwide [16, 17].

Challenges hindering objective, evidence-based decision-making in malaria control can be addressed by developing new policy tools to enable policy makers from different sectors systematically evaluate the probable health, economic and environmental consequences of different vector and disease management strategies [10]. The objective of the present study was to assess the factors that currently influence malaria control decisions in Kenya, Uganda and Tanzania, as a crucial first step in the participatory development of a malaria decision analysis support tool (MDAST) for promoting multi-sectoral evidence-based policy-making at the national malaria control programme (NMCP) level [18].

## Methods

The study involved stakeholder surveys conducted in the three East African countries, Kenya, Uganda and Tanzania between March and August 2010. The surveys mainly sought to answer the following specific questions: 1) What is the level of collaboration between the agency mainly responsible for malaria control and policymakers from various sectors in generating, disseminating, and applying evidence relevant to malaria control policy?, 2) Do policymakers consider a range of health, environmental, social, and economic factors in formulating policy?, and 3) Are malaria control decisions informed by evidence from a variety of sources?

### Survey administration

The survey respondents in each country were drawn from a non-random purposeful sample of stakeholders. The study targeted individuals in health and other government ministries, non-governmental organizations (NGOs), universities and research institutes whose policy decisions and actions are likely to have impact on the status of malaria or influence malaria control decision-making in the respective countries. The primary sectors represented were those responsible for health, agriculture and environment issues. The in-country project leads, who represented key national malaria policy-making institutions, used their professional networks to identify and contact relevant high-level stakeholders from government, academic, and non-profit sectors. Specific individuals from the relevant stakeholder organizations were considered to be generally knowledgeable regarding malaria and its control.

A draft version of the survey questionnaire to be used in the study was first pre-tested among participants from health and other sectors attending a project inception meeting in Nairobi in March 2010. The draft was then revised based on a number of suggestions for improving the questions, wording and format. The survey questionnaire was administered to respondents in hard-copy for completion by hand. The final survey instrument is accessible at the MDAST project website [19]. The gathered information was entered into a Microsoft Access database.

Consecutive sections of the survey questionnaire were administered as follows: Section I- professional background of stakeholder respondents; Section II - national malaria control decision making; Section III- criteria and indicators for policy decisions; and Section IV - malaria control. Section IV contained the following sub-sections: vector control (ITNs/LLINs, IRS with pyrethroids or DDT, and larviciding); treatment (ACT, IPTp/IPTi); and diagnosis (RDTs, microscopy, clinical diagnosis). A total of 97 questionnaires were conducted and analysed. There were 31 surveys completed by participants in Tanzania, 33 from Uganda, and 33 from Kenya. Prior to survey administration, ethical approval for the study was granted by the Institutional Review Board at Duke University.

### Data analysis

Summary measures of the survey data were aggregated across all three project countries as the individual country sample sizes were too small for meaningful comparison across countries. The parameters of focus included summary statistics on various factors, objectives, indicators and risks. Where applicable, importance values were assigned for each category using a 5-point Likert scale whereby: 1 = not important; 5 = very important [20].

## Results

### Professional background of stakeholder respondents

Respondents were asked to self-identify the type of organization and sector they worked in. They were asked to choose only one type of organization and one sector from the category lists provided (respondents could also write in a different response, but these responses were tallied separately under the “other” category except where readily assignable to an existing category). Respondents who chose more than one category were reported in only one category, randomly assigned from among the categories the respondent selected. One participant did not respond to these questions. More respondents worked for a government entity (49%) than for any other type of organization across all countries. The second most represented organization type among respondents was “University/Research Institution” (22%). Less represented types of organization were “NGO/Civil Society/Faith-based Organization”, “Donor Agency” and “Other”, with their respondents respectively constituting 9%, 4% and 16% of the respondents. As regards the actual professional sector, “Health” had the highest representation among the respondents (71%). Other represented sectors respectively comprised of “Agriculture” (10%), “Environment” (7%), “Education” (5%), “Finance/Trade” (1%) and “other” 5%. The breakdown of self-reported organizational and sector affiliation by country is contained in Table 1.

**Table 1:** **Organization and sector affiliation of respondents by country**

	Overall		Tanzania		Uganda		Kenya	
Organization	N	%	N	%	N	%	N	%
Government	47	49%	15	48%	15	47%	17	52%
University/Research Institution	21	22%	8	26%	8	25%	5	15%
Donor Agency	4	4%	3	10%	0	0%	1	3%
NGOs/Civil Societies/Faith-based Organization	9	9%	3	10%	3	9%	3	9%
Other	15	16%	2	6%	6	19%	7	21%
**Total**	96	100%	31	100%	32	100%	33	100%
**Sector**	**N**	**%**	**N**	**%**	**N**	**%**	**N**	**%**
Health	68	71%	23	74%	24	75%	21	64%
Agriculture	10	10%	2	6%	3	9%	5	15%
Environment	7	7%	3	10%	1	3%	3	9%
Education	5	5%	1	3%	3	9%	1	3%
Finance/Trade	1	1%	0	0%	1	3%	0	0%
Other	5	5%	2	6%	0	0%	3	9%
**Total**	96	100%	31	100%	32	100%	33	100%

### National malaria control decision-making

**Figure 1:**
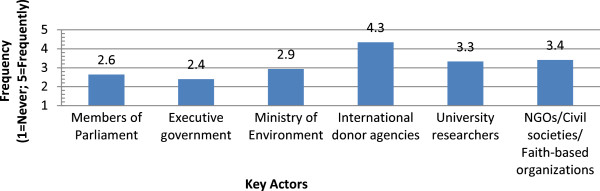
**Meetings with agency with main responsibility for malaria control policies.**

A series of survey questions addressed stakeholder perspectives on national malaria control decision-making. This section gathered information from stakeholder participants in each country on factors taken into consideration in determining national malaria control policies. This section also asked respondents to juxtapose the current situation and the ideal situation in their respective countries with regards to the importance of various factors influencing national malaria control policies. Figure 1 summarizes stakeholder responses on the frequency with which the main agency responsible for national malaria control policy meets with six selected actors. Aggregated across countries, stakeholders reported that the main agency for malaria control policy met with international donor agencies significantly more often than with other key actors (4.3 on the 5-point Likert scale, or nearly frequently). Other actors with which the main agency was perceived to meet with occasionally or more than occasionally were NGOs/Civil Societies/Faith-based organizations (3.4, or more than occasionally), university researchers (3.3, more than occasionally) and the Ministry of Environment (2.9, near occasionally). Stakeholders reported that the agency mainly responsible for malaria control policies met with members of parliament (2.6) and the executive government (2.4) less than occasionally.

**Figure 2:**
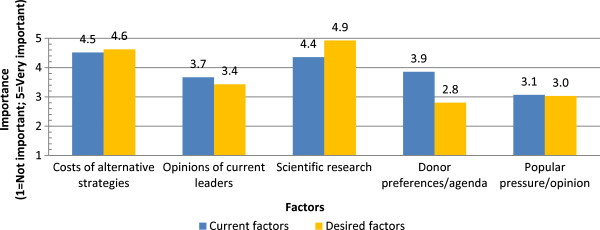
**Factors determining national malaria control policies.**

Figure 2 compares the average ratings of selected factors in determining national malaria control policies in respondents’ countries, as perceived for both the current situation and a desired ideal situation. Overall, respondents reported that the cost of alternative control strategies was currently a very important factor in determining policy, as they felt should be the case in an ideal scenario. However, there were factors for which respondents assigned different levels of current and desired importance in the policy-making process; overall, responses supported giving more importance to scientific research compared to the current scenario, but indicated that in an ideal scenario, less importance should be given to donor preferences and agendas. The respondents were also asked to list alternative factors, other than those in the survey, which they deemed important in the decision-making process. Among those factors listed as currently being important in this process were: community considerations (mentioned by two respondents), current disease trends (one respondent), global action (one respondent), the opinions of WHO (one respondent), politics (one respondent), sustainability and consistency (one respondent) and operational feasibility (one respondent). Other (unlisted) factors which respondents thought should be important in determining national malaria control policies were community considerations (three respondents), export markets (one respondent), feasibility (one respondent), sustainability and consistency (one respondent), demographic trends (one respondent), media (one respondent), safety profile interventions (one respondent), disease trends (one respondent), and regional action (one respondent).

### Criteria and indicators for policy decisions

**Figure 3:**
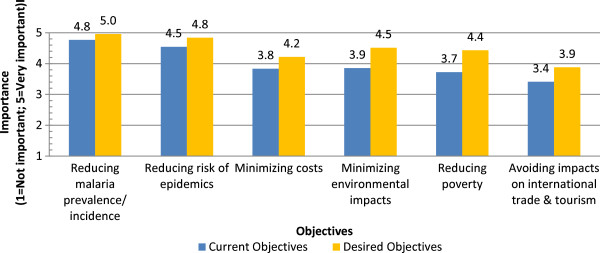
**Objectives for malaria control policies.**

In this section of the survey stakeholder participants in each country were asked to juxtapose the current situation and the ideal situation in their respective countries with regards to the importance of various objectives influencing national malaria control policies. The section also asked for stakeholder input on the importance of various indicators and risks with regards to malaria control activities and decision-making. Figure 3 compares the average ratings according to a five-point Likert scale aggregated across all project countries for both the current situation and the desired situation. The juxtaposition revealed a number of interesting observations: overall, the average importance value assigned to objectives was consistently higher in the desired situation than in the current situation. The largest gap in the average importance value between the current and desired situation was for the objective “reducing poverty” (3.7 and 4.4, respectively, a gap of 0.7 on the five-point Likert scale); The smallest gap in the average importance value between the current and desired situation was for the objective “reducing malaria prevalence/incidence” (a gap of 0.2). As regards a listing of alternative objectives, the respondents generally suggested the following as being important: reducing mortality, developing eco-friendly interventions, and fulfilling the objectives of the Abuja declaration on malaria.

**Figure 4:**
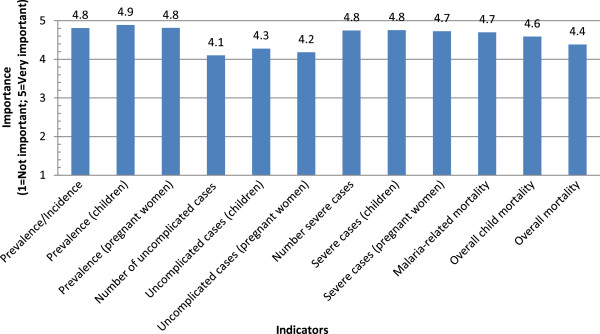
**Importance of indicators for policymakers.**

**Figure 5:**
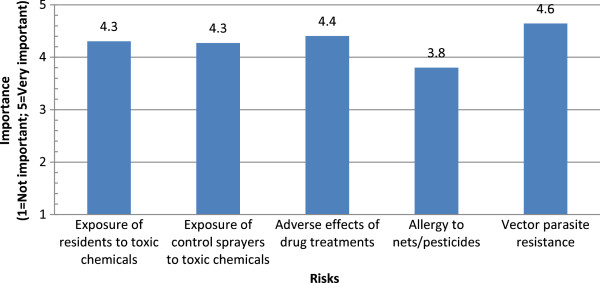
**Risks for human health impacts of malaria control activities.**

Figure 4 shows the average importance given to each of the selected indicators of the human health impact of malaria in policymakers’ considerations according to stakeholder respondent perceptions. Many of the indicators were ranked very high, at 4.8 or above, near the 5.0 value for “very important” on the Likert scale (overall prevalence/incidence, prevalence/incidence among children, prevalence among pregnant women, number of severe cases, and number of severe cases among children). The indicator ranked as least important (number of uncomplicated cases) still had a high value of importance on the Likert scale (4.1).Figure 5 shows the average importance given to each of the selected risks for human health impacts of malaria control activities. Overall, all risks ranked above 3.0 (neutral) on the Likert scale for importance. The risk ranked as having the highest importance was “vector parasite resistance” (4.6 on the Likert Scale, nearly very important) while the lowest was “allergy to nets/pesticides” (3.8 on the Likert Scale).

### Malaria control

A series of questions in the survey asked stakeholder participants to evaluate the importance of a range of selected factors when deciding on the use of specific malaria control strategies. The questions were separated into three sub-sections namely: vector control, treatment and, diagnosis.

#### ITNs versus LLINs

**Figure 6:**
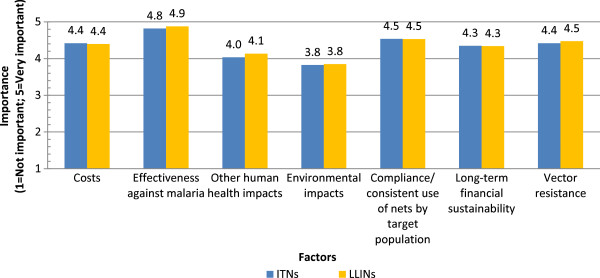
**Deciding on the use of nets (ITNs and LLINs).**

Under vector control, respondents were asked to compare the importance of a range of factors in deciding on the use of insecticide-treated nets (ITNs) i.e. that have to be periodically retreated, or long-lasting insecticide-treated nets (LLINs). Figure 6 compares the average importance values in the decision-making process assigned to the factors. Overall, the average importance values were very nearly similar for a given factor between ITNs and LLINs (never more than a 0.1 point difference on the Likert scale). This suggests that the importance of a factor does not vary based on the type of net intervention being considered. The factor ranked of highest importance was “effectiveness against malaria” (4.8 and 4.9 for ITNs and LLINs, respectively). The factor with the lowest importance value (environmental impacts, 3.8 for both net interventions) was, however, still ranked above neutral in importance.

#### IRS with pyrethroids or DDT

**Figure 7:**
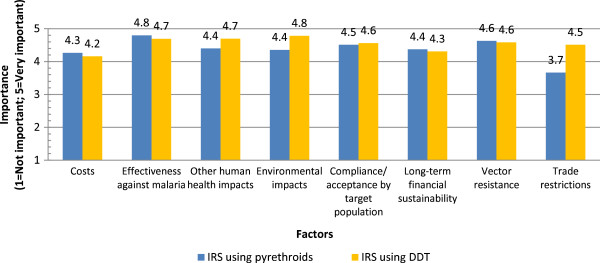
**Deciding on the use of IRS (pyrethroids or DDT).**

Respondents were also asked to compare the importance of a range of factors in deciding on the use of IRS using pyrethroids or DDT. Figure 7 compares the aggregate average importance values in the decision-making process assigned to the factors. Overall, many of the aggregate average importance values were fairly similar for a given factor between using pyrethroids or DDT. However, the importance of trade restrictions was ranked higher for DDT (4.5) than for pyrethroids (3.7). “Effectiveness against malaria” had high importance values for both pyrethroids (4.8) and DDT (4.7). A high importance value for DDT was also assigned to environmental impacts (4.8), compared to a lower value of 4.4 for the importance of environmental impacts with regards to pyrethroids.

The respondents were also queried about additional factors they considered important in deciding whether to use pyrethroids for IRS. Biodegradable nature of pyrethroids, impacts on other disease-transmitting vectors, and the residual effect of pyrethroids were given as important factors.

#### Larvicides

**Figure 8:**
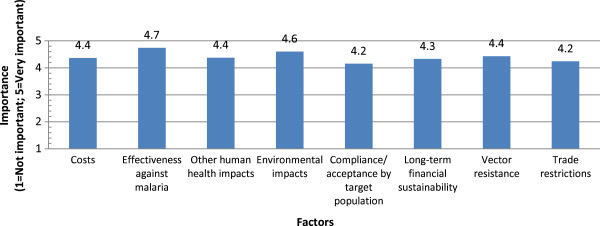
**Deciding on the use of larvicides.**

Respondents were asked to compare the importance of a range of factors influencing the use of larvicides. Figure 8 shows the average importance values assigned by respondents for the various factors. Overall, all specified factors were rated as above 4.0 in importance. The highest-rated factor was “effectiveness against malaria” (4.7) while the lowest-rated factors were “compliance/acceptance by target population” and “trade restrictions” (both 4.1).

#### Treatment

**Figure 9:**
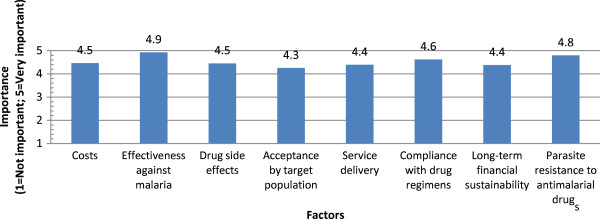
**Deciding on the use of ACT.**

**Figure 10:**
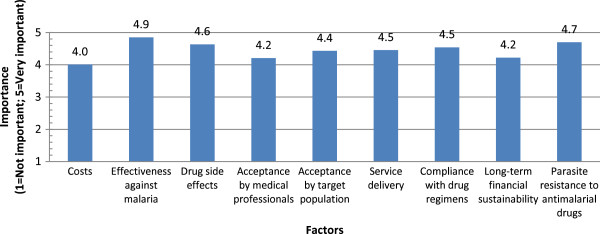
**Deciding on the use of IPTp and IPTi.**

Regarding treatment, respondents were asked to compare the importance of a range of factors in deciding on the use of artemisinin combination therapy (ACT) for malaria treatment. Figure 9 shows the average importance values assigned by the respondents. Overall, all specified factors were rated as above 4.0 in importance. The lowest-rated specified factor was “acceptance by target population” (4.3). The highest-rated factor was “effectiveness against malaria” (4.9).Figure 10 shows the average importance values assigned by respondents for a range of factors with regards to the use of intermittent preventative treatment for pregnant women and infants (IPTp and IPTi). Overall, all factors were rated as at least 4.0 in importance. The lowest-rated factor was “costs” (4.0). The highest-rated factor was “effectiveness against malaria” (4.9).

#### Diagnosis

**Figure 11:**
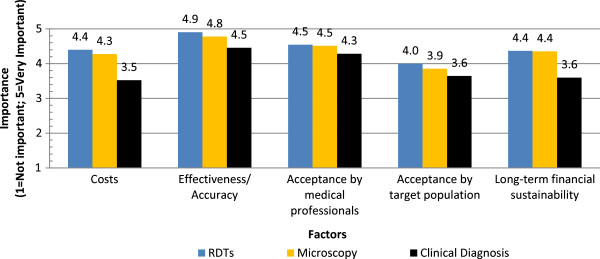
**Diagnostic priorities for malaria.**

In regard to diagnosis, respondents were asked to compare the importance of a range of factors influencing the use of different diagnostic strategies (RDTs, Microscopy, Clinical diagnosis). Figure 11 shows the average importance values assigned by respondents for the various factors. Overall, all specified factors were rated as at or above 3.5 in importance for all diagnostic strategies. The lowest-rated specified factor was “costs” for clinical diagnosis (3.5). The highest-rated factor was “effectiveness/accuracy” for RDTs (4.9). Respondents ranking acceptability by the target population as a less important factor across all diagnostic methods relative to the other factors may think it unlikely to be an issue (i.e., they consider it to be aless important factor in decision-making because they believe community acceptability of the diagnostic methods to already be high). Respondents also expressed that quality control mechanisms for these diagnostics, and knowledge as to their use were other key considerations.

## Discussion

Results aggregated across countries in the present study highlighted, among other observations, a belief that donor preferences and agendas were exerting too much influence on malaria policies in the project countries. This observation may appear counterintuitive considering that malaria has significantly reduced in Africa during the past decade primarily due to a dramatic increase in donor funding combined with domestic mobilization of resources [21, 22]. However, the contradiction could be due to a concern that international financing for malaria control is unsustainable in view of economic recession conditions and consequent austerity measures among certain donor countries. The respondents’ perceptions were in agreement with similar views from recent analyses of resource flows for malaria, which provide ample evidence of overreliance on donor funding [21].

The apprehension that external funding can eventually be counterproductive if not carefully balanced with a corresponding mobilization of a country's own resources was corroborated by many respondents’ other view that the agency in charge of malaria control did not adequately engage members of parliament. This latter result, as is indeed generally the case for the whole of this qualitative study, needs to be interpreted with caution considering that certain responses by respondents (29%) not working directly within the health sector may have been speculative to a varying degree, and not necessarily informed by prior experiences or insights.

Nevertheless, it is worth noting that the involvement of members of parliament and the executive is in many cases crucial for providing the political support and influencing the legislation needed to sustain malaria control programmes on a long-term basis. For instance, legislation leading to a waiver of taxes on insecticide-treated nets and the positive impact it has had towards up-scaling of the intervention might be difficult to effect unless members of parliament are fully engaged [23]. The donor influence situation in Kenya, Tanzania and Uganda was, in this study, similar to that in Mozambique, but different from that in South Africa where the government through its department of health is known to be relatively independent in making its malaria control decisions [24]. The situation in South Africa is primarily attributed to the country’s ability to financially support its malaria control programme without having necessarily to resort to external donor funding. It’s no wonder then that South Africa’s goal of eliminating malaria by 2018 appears realistic due to solid political support and the domestic mobilization of resources [25]. The perception regarding donors’ influence in malaria policy decisions has been supported by a previous observation in Tanzania that 85% of the country’s national malaria control programme’s strategic plan has been funded by external donors [26]. Such a scenario has been described to result in donor dependency and, should the support disappear, countries would be hard-pressed to finance their ongoing malaria control programmes [27].

A second important result of the survey was in connection with the perception by the respondents that some of the relevant objectives of malaria control were not being given enough consideration during decision-making. This was particularly the case with regard to reducing poverty and minimizing environmental impacts of vector control interventions. Regarding other objectives, it was notable that scientific research was accorded high importance by respondents. Nevertheless, the respondents felt that this factor required even greater attention than was currently the case in spite of the relatively high value of importance currently attached to it. This result suggested that evidence-based decision-making was highly valued by the respondents. Several examples of using strong scientific evidence for critical decisions regarding selection of either vector control interventions or new first line drugs for malaria treatment have been reported in the past [28].

The development and implementation of the survey had certain other limitations which should be considered in interpreting the results. Among these was the fact that during the pre-testing and subsequent administration of the survey questionnaire, respondents from both the health and non-health sectors were mainly technical staff, involved in providing policy advice, but not necessarily in making the final policy decisions themselves. A similar situation where interviews have not included political figures like ministers and presidents, who are sometimes the ones who directly make policy in certain African countries, has recently been described in a study investigating factors influencing health policy entrepreneurs in West Africa [29]. Nevertheless, the West African study importantly noted that the inputs of government officials who are charged with giving technical advice significantly influence the final decision-making.

## Conclusion

This study investigated the views of malaria control stakeholders about influencing factors and objectives of malaria control policy across several East African countries. Malaria control decisions in Kenya, Uganda and Tanzania are effected based on a general assessment of health and environmental impacts and cost implications of different intervention strategies. Further engagement of government legislators and other policy makers is needed in order to increase resource flows from domestic sources, reduce donor dependence, sustain interventions and consolidate current gains in malaria.

Overall, the study led to a greater understanding of the perspectives of stakeholders from different sectors among three East African countries regarding the relevance of a wide range of factors and objectives considered as being important in determining malaria control strategies. The factors included costs of interventions, effectiveness, human health and environmental impacts, compliance or acceptance, financial sustainability, and vector resistance. The stakeholder information helped fill critical knowledge gaps towards completing the development of MDAST [18]. It is anticipated that the MDAST will serve as a useful and practical tool for assisting policy-makers in their implementation of a multi-sectoral approach to malaria as has been strongly advocated by WHO and RBM [30].

## References

[1] WHO (2013). World Malaria Report.

[2] Teklehaimanot A, Mejia P (2008). Malaria and poverty. Ann NY AcadSci.

[3] Newman RD (2012). Relegating malaria resurgences to history. Malar J.

[4] WHO (2012). Global Plan for Insecticide Resistance Management in Malaria Vectors.

[5] Nanyunja M, Orem JN, Kato F, Kaggwa M, Katureebe C, Saweka J (2011). Malaria treatment policy change and implementation: the case of Uganda. Malaria Res Treat.

[6] Dondorp AM, Nosten F, Yi P, Das D, Phyo AP, Tarning J, Lwin KM, Ariey F, Hanpithakpong W, Lee SJ, Ringwald P, Silamut K, Imwong M, Chotivanich K, Lim P, Herdman T, An SS, Yeung S, Singhasivanon P, Day NPJ, Lindegardh N, Socheat D, White NJ (2009). Artemisinin resistance in *Plasmodium falciparum* malaria. N Eng J Med.

[7] Barat LM (2006). Four malaria success stories: how malaria burden was success fully reduced in Brazil, Eritrea, India and Vietnam. Am J Trop Med Hyg.

[8] Leach-Kemon K, Chou DP, Schneider MT, Tardif A, Dielman JL, Brooks BPC, Hanlon M, Murray CJL (2012). The global financial crisis has led to a slowdown in growth of funding to improve health in many developing countries. Health Aff.

[9] Sachs J, Malaney P (2002). The economic and social burden of malaria. Nature.

[10] Kramer R, Dickinson KL, Johnson RW, Anderson RM, Fowler VG, Miranda ML, Mutero CM, Saterson KA, Wiener JB (2009). Using decision analysis to improve malaria control policy making. Health Policy.

[11] Bouwman H, Van den Berg H, Kylin H (2011). DDT and malaria prevention: addressing the paradox. Environ Health Perspect.

[12] Biscoe M, Mutero CM, Kramer R (2005). Current Policy and Status of DDT use for Malaria Control in Ethiopia, Uganda, Kenya and South Africa. Working Paper 95.

[13] Mabaso MLH, Sharp B, Lengeler C (2004). Historical review of malaria control in southern Africa with an emphasis on the use of indoor residual house-spraying. Trop Med Int Health.

[14] Maharaj R, Mthembu DJ, Sharp BL (2005). Impact of DDT reintroduction on malaria transmission in KwaZulu-Natal. S Afr Med J.

[15] Chanda E, Masaninga F, Coleman M, Sikaala C, Katebe C, MacDonald M, Kumar S, Baboo KS, Govere J, Manga L (2008). Integrated vector management: the Zambian experience. Malar J.

[16] Aneck-Hahn NH, Schulenburg GW, Bornman MS, Farias P, de Jager C (2007). Impaired semen quality associated with environmental DDT exposure in young men living in a malaria area in the Limpopo Province. South Africa J Androl.

[17] De Jager C, Farias P, Barraza-Villarreal A, Avila MH, Ayotte P, Dewailly E, Dombrowski C, Rousseau F, Sanchez VD, Bailey JL (2006). Reduced seminal parameters associated with environmental DDT exposure and p, p’-DDE concentrations in men in Chiapas, Mexico: a cross-sectional study. J Androl.

[18] WHO - AFRO (2013). Malaria Decision Analysis Support Tool (MDAST): Final Report.

[19] **MDAST**. http://sites.duke.edu/mdast/surveys/

[20] Burns A, Burns R (2008). Basic Marketing Research.

[21] Pigott DM, Atun R, Moyes CL, Hay SI, Gething PW (2012). Funding for malaria control 2006–2010: a comprehensive global assessment. Malar J.

[22] Chanda E, Mukonka VM, Kamuliwo M, Macdonald MB, Haque U (2013). Operational scale entomological intervention for malaria control: strategies, achievements and challenges in Zambia. Malar J.

[23] Alilio M, Mwenesi H, Barat ML, Payes RM, Prysor-Jones S, Diara M, McGuire D, Shaw W (2007). Broken promise? Taxes and tariffs in insecticide-treated mosquito nets. Am J Trop Med Hyg.

[24] Cliff J, Lewin S, Woelk J, Fernandes B, Mariano A, Sevene E, Daniels K, Matinhure S, Oxman A, Lavis J (2010). Policy development in malaria vector management in Mozambique, South Africa and Zimbabwe. Health Policy Plan.

[25] Coetzee M, Kruger P, Hunt RH, Durrheim DN, Urbach J, Hansford CF (2013). Malaria in South Africa: 110 years of learning to control the disease. S Afr Med J.

[26] Ministry of Health and Social Welfare (United Republic of Tanzania) (2008). Medium Term Malaria Strategic Plan 2008–2013.

[27] Mboera LEG, Mazigo HD, Rumisha SF, Kramer R (2013). Towards malaria elimination and its implication for vector control, disease management and livelihoods in Tanzania. Malaria World J.

[28] Shretta R, Omumbo J, Rapuoda B, Snow RW (2000). Using evidence to change antimalarial drug policy in Kenya. Trop Med Int Health.

[29] Torbica A, De Allegri M, Belemsaga DY, Medina-Lara A, Ridde V (2013). What Factors Influence Health Policy Entrepreneurs in West Africa.

[30] RBM: *Multisectoral Action Framework for Malaria 2013*. Geneva: Roll Back Malaria; Available online: http://www.rbm.who.int/docs/2013/Multisectoral-Action-Framework-for-Malaria.pdf

